# Characteristics of Japanese encephalitis virus infection in NCG-hSTAT2^+/+^ mice: a novel model for studying neurological symptoms and immune response

**DOI:** 10.1242/dmm.052431

**Published:** 2025-11-18

**Authors:** Caiqin Zhang, Yiran Li, Yifan Zhou, Yong Zhao, Pengpeng Wu, Bing Bai, Yifan Ma, Jing Qin, Han Meng, Yangchao Dong, Changhong Shi

**Affiliations:** ^1^ Division of Cancer Biology, Laboratory Animal Center, The Fourth Military Medical University, Xi'an, Shaanxi 710032, China; ^2^ School of Basic Medical Sciences, Medical College of Yan'an University, Yan'an, Shaanxi 716000, China; ^3^ First School of Clinical Medicine, Gansu University of Traditional Chinese Medicine, Lanzhou, Gansu 730030, China; ^4^ Department of Microbiology & Pathogen Biology, School of Basic Medical Sciences, The Fourth Military Medical University, Xi'an, Shaanxi 710032, China

**Keywords:** Japanese encephalitis virus (JEV), Interferon, STAT2, Humanized model, Neurological symptoms

## Abstract

Japanese encephalitis virus (JEV), a leading cause of viral encephalitis in Asia and the Western Pacific, is regulated by type I interferon (IFN) signaling pathway, in which STAT2 is critical. However, the exact role of STAT2 in JEV-mediated IFN evasion remains unclear. Existing murine models of JEV infection predominantly employ high viral titers to induce encephalitis and primarily use immunocompetent or IFN receptor-deficient mice, limiting their use to study the IFN evasion mechanisms of JEV. To address this, we developed a humanized STAT2 mouse model (NCG-hSTAT2^+/+^) and infected it with 10³ PFU of JEV-p3. These mice exhibited severe encephalitis resembling clinical human infections, characterized by elevated viral load, and increased proinflammatory cytokines. Especially, they presented typical neurological symptoms, such as activated astrocytes and distinct neuropathological changes. This suggests that NCG-hSTAT2^+/+^ mice exhibit higher susceptibility to JEV and more-severe neurological symptoms, which is consistent with the clinical manifestations observed in human patients. This mouse model significantly advances the study of JEV pathogenesis, the therapeutic evaluation of this infection and the role human STAT2 has in neuroinvasion and immune evasion.

## Teaser

**Summary:** NCG-hSTAT2+/+ mice exhibit higher susceptibility to JEV and more severe neurological symptoms. This model significantly advances the study of JEV pathogenesis, therapeutic evaluation, and understanding the role of human STAT2 in neuroinvasion and immune evasion.

## INTRODUCTION

Japanese encephalitis virus (JEV), a mosquito-borne Flavivirus related to Dengue, Zika and West Nile viruses, threatens global health with ∼68,000 annual cases (Sahu et al., 2022). Infections range from mild fever to severe neurological complications, like seizures, paralysis and altered consciousness (Miyake, 1964; Solomon, 2003). Due to varied symptoms and limited treatments, better therapies are urgently needed.

The innate immune system is the primary defense against JEV infection in humans, initiating immediate protective mechanisms against pathogen invasion. Type I interferons (IFNs) are a crucial regulator of viral infections in the innate immune system (Jensen and Thomsen, 2012). Upon binding to their receptors, type I IFNs activate the Janus kinase (JAK)-signal transducer and activator of transcription (STAT1/2) signaling pathway, which regulates expression of downstream interferon-stimulated genes (ISGs) (Doly et al., 1998; Haller et al., 2006). Signal transducer and activator of transcription 2 (STAT2) is a key molecule in the IFN pathway; however, its function differs between humans and mice. The non-structural protein 5 (NS5) of Dengue and Zika viruses (DENV and ZIKV, respectively) mediates the degradation of STAT2 in human cells to promote the replication of viruses. Murine STAT2 is resistant to degradation by the NS5 proteins of DENV and ZIKV, rendering mice inherently more resistant to these flaviviruses (Morrison and García-Sastre, 2014; Grant et al., 2016). Thus, human STAT2 knock-in (hSTAT2-KI) C57BL/6 mice have previously been used to enhance susceptibility to ZIKV infection (Kim et al., 2024; Yang et al., 2024). Currently, the immune escape mechanisms of JEV against IFN pathways remain incompletely understood, i.e. the mechanism by which JEV degrades STAT2 is still unknown. Based on the role of STAT2 in DENV and ZIKV immune evasion, we hypothesized that JEV employs a similar strategy and generated a mouse model expressing the human *STAT2* gene in order to study JEV infection.

Mice strains, such as C3H/He, C57BL/6, BALB/c and DBA/2, are the most widely used animal models for studying replication of JEV and neuropathogenesis of Japanese encephalitis (JE) (Bhowmick et al., 2007; Ooi et al., 2008; Kimura et al., 2010). However, these mouse models display varying sensitivity to JEV. In studies of JEV infection that have used C57BL/6 and C3H/He mice, a high viral inoculum – i.e. 10^5^–10^6^ plaque-forming units (PFU) – was typically required and the onset of significant encephalitis symptoms was notably delayed. It is worth noting that C57BL/6 and C3H/He mice possess fully functioning immune systems. By contrast, adaptive immune responses are triggered when pathogens evade the innate immune system and reach threshold levels of antigen production in vertebrates. It was found that mice lacking B cells exhibit increased susceptibility to flaviviruses (Charlier et al., 2002; Halevy et al., 1994), and another study underscored the importance of CD4-positive and CD8-positive T cells in resisting JEV infection (Murali-Krishna et al., 1996). Owing to the complexity of infection and immunity, model mice with an intact immune system might not be conducive to mechanistic studies of the IFN signaling pathway. Although interferon alpha and beta receptor subunit 1 (IFNAR1) knockout (KO) mice have been widely used to study JEV infection, they only display peripheral pathological responses and do not exhibit neurological symptoms after infection with JEV (Liu et al., 2022).

To investigate the impact of STAT2 on JEV infection and to better simulate severe human neurological symptoms, we used CRISPR-Cas9 technology and severely immune-deficient NCG mice (Xu et al., 2022) to construct a humanized STAT2 gene mouse model expressing the human *STAT2* gene (hereafter referred to as NCG-hSTAT2^+/+^ mice). When infected with a lower dose of JEV (10³ PFU), this NCG-hSTAT2^+/+^ mouse model exhibited distinctive immune responses and enhanced susceptibility compared to other mouse strains. Particularly, NCG-hSTAT2^+/+^ model mice were able to simulate typical neurological symptoms and a completely humanized IFN immune response, providing a new JEV research experimental tool.

## RESULTS

### Construction and identification of NCG-hSTAT2^+/+^ mice

**Fig. 1.**
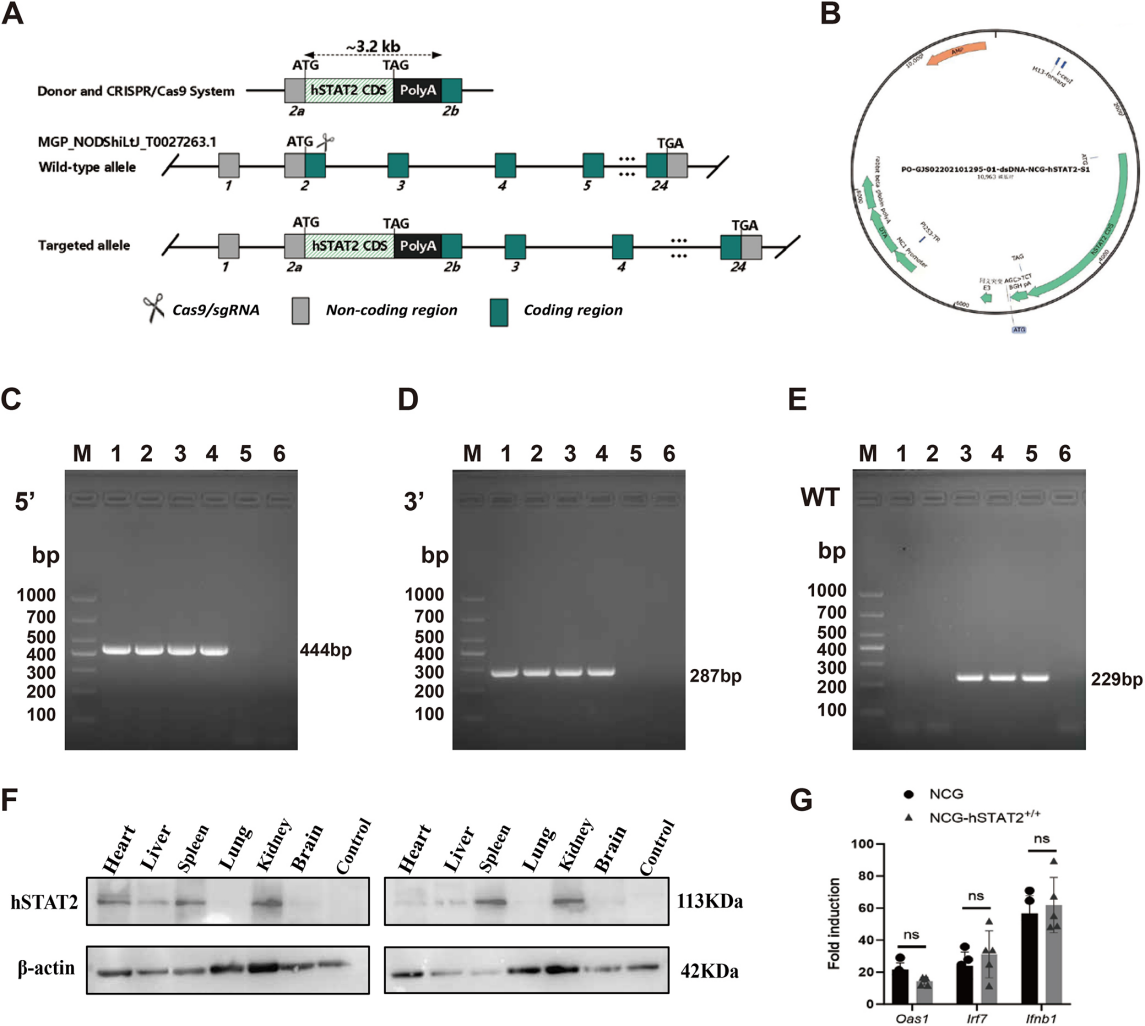
**Construction strategy and identification of NCG-hSTAT2^+/+^ mice.** (A) Schematic of the strategy used to express the human *STAT2* gene (hSTAT2) in mouse. CDS, coding DNA sequence. (B) Expression vector of the hSTAT2 gene. (C) Agarose gel electrophoresis image of PCR products, showing positive staining for a specific 444 bp region within the 5′ end of the hSTAT2 gene (lanes 1–4); lanes 5 and 6 were negative. (D) Agarose gel electrophoresis image of PCR products, showing positive staining for a specific 287 bp region within the 3′ end of the hSTAT2 gene (lanes 1–4); lanes 5 and 6 were negative. (E) Agarose gel electrophoresis image of PCR products. Positive staining for a specific 229 bp fragment of the mouse STAT2 gene is shown in lanes 3–5, whereas lanes 1 and 2 stained negative. M, Marker. Lanes 1-5 contain PCR products from DNA samples obtained from mice 1-5, respectively; lane 6 is the ddH_2_O control. 1st and 2nd mice are named NCG-hSTAT2^+/+^ with homozygous target genes. 3rd and 4th mice are named NCG-hSTAT2^+/−^ with heterozygous target genes. The 5th mouse is named NCG because of no hSTAT2 gene. (F) Western blot analysis of organ samples obtained from NCG-hSTAT2^+/+^ mice further confirmed the presence of hSTAT2 protein in all organs, with only expression in lung being substantially reduced. Control indicates kidney sample from NCG mice. (G) Quantification of induction of interferon-stimulated genes (i.e. *Oas1*, *Irf7*, *Ifnb1*) in WT and NCG-hSTAT2^+/+^ mice after intraperitoneal administration of Poly (I:C)(HMW). Fold induction of the above genes in the spleen of NCG and NCG-hSTAT2^+/+^ (*n*=5) was analyzed 6 h later, showing that levels of *Oas1*, *Irf7* and *Ifnb1* mRNA were comparable and not statistically different. Nd, not statistically different. Error bars indicate the mean ±standard deviation.

NCG-hSTAT2 mice were produced using CRISPR-Cas9 technology. To express the human STAT2 gene in NCG mice, the insertion expression mode diagram of hSTAT2 gene (Fig. 1A) and expression vector diagram (Fig. 1B) were designed. Mouse offspring was identified by PCR. As shown in Fig. 1C-E, 1st and 2nd mice were named NCG-hSTAT2^+/+^ with homozygous target genes, 3rd and 4th mice were named NCG-hSTAT2^+/−^ with heterozygous target genes, the 5th mouse was named NCG as it does not express the hSTAT2 gene. The organs of NCG-hSTAT2^+/+^ mice were analyzed by western blotting. The hSTAT2 protein was variably expressed in NCG-hSTAT2^+/+^ mouse tissues (Fig. 1F). Following intraperitoneal administration of high molecular weight (HMW) polyinosinic-polycytidylic acid [Poly (I:C)(HMW)], splenic induction of ISGs (i.e. *Oas1*, *Irf7*, *Ifnb1*) was confirmed in NCG-hSTAT2^+/+^ mice 6 h post injection. Expression levels of IFN were found to be indistinguishable from those in WT NCG mice (Fig. 1G), indicating that expression of hSTAT2 did not block the IFN signaling pathway.

### High susceptibility to JEV in NCG-hSTAT2^+/+^ mice

**Fig. 2.**
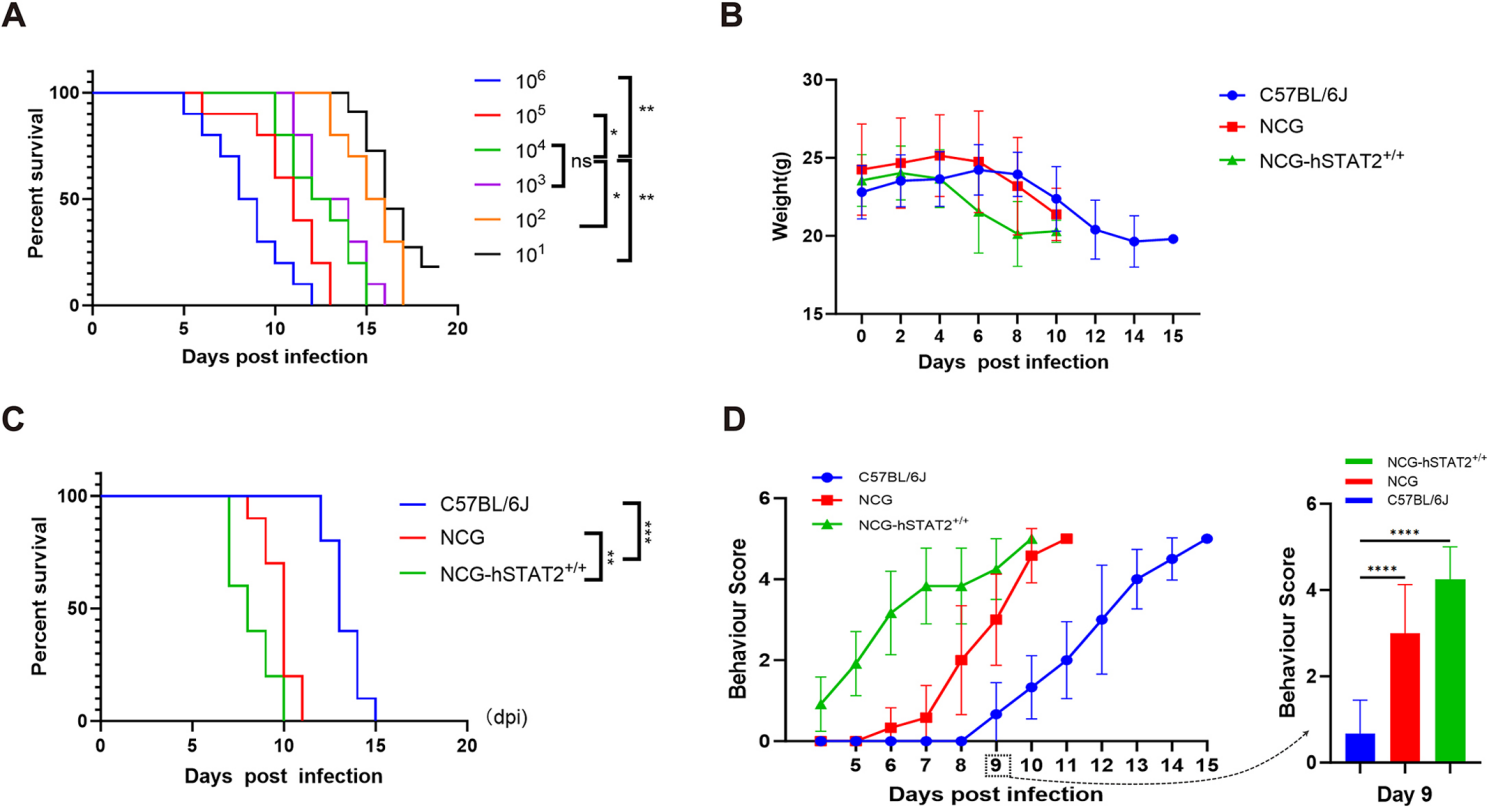
**NCG-hSTAT2^+/+^ mice are more susceptible to JEV at 10^3^ PFU than C57BL/6J and NCG mice.** (A) Survival curves of C57BL/6J mice infected with different doses of JEV-p3, showing that 1×10^3^ PFU was the optimal infectious dose. (B) Body weight changes of JEV-infected C57BL/6J, NCG and NCG-hSTAT2^+/+^ mice. (C) Kaplan–Meier curves showing the survival of C57BL/6J, NCG and NCG-hSTAT2^+/+^ mice after JEV infection. (D) Left: Plotted are clinical sign scorings of the three JEV-infected C57BL/6J, NCG or NCG-hSTAT2^+/+^ mice, showing that symptom scores differed markedly between the three groups, with symptoms in the NCG-hSTAT2^+/+^ cohort scoring highest. Right: Statistical analysis comparing the scoring of clinical signs between these mice on 9 dpi. Error bars indicate the ±standard error. *n*=10 per group of mice (A–D). Statistical significance was calculated using ANOVA, with *P<0.05, **P<0.01, ***P<0.001, ****P<0.0001. ns, not significant.

To investigate the potential involvement of hSTAT2 in JEV-infected disease stress conditions, we inoculated JEV into the footpad of NCG-hSTAT2^+/+^ mice to simulate the infection process of natural mosquito bites as described by Yun and Lee (2014). To determine the appropriate titer for JEV-p3 strain infection, a series of JEV diluents were injected into C57BL/6J mice. The mortality rate of mice increased with increased viral dose, and the earliest deaths occurred 5 days post infection (dpi) following 10^5^–10^6^ PFU. At 20 dpi, one mouse was still alive in the 10^1^ PFU group. However, the percent survival was not significantly different between at doses of 10^3^ PFU and 10^4^ PFU (Fig. 2A, *P*<0.05, *P*<0.01). To investigate the effect of JEV after infection at a lower dose, an incubation dose of 10^3^ PFU/mouse was chosen for infection in all subsequent experiments.

C57BL/6J, NCG and NCG-hSTAT2^+/+^ mice injected with JEV-p3 (10^3^ PFU) into their distal footpads and observed for 15 dpi until death. The body weight of each mouse in each group was recorded daily (Hatzipetros et al., 2015). and data analysis revealed that NCG-hSTAT2^+/+^ mice started losing weight on 3 dpi and NCG mice on 5 dpi. The body weight of C57BL/6J mice increased during the first 8 dpi and began to decrease on 9 dpi and thereafter (Fig. 2B). Moreover, NCG-hSTAT2^+/+^ mice showed a series of symptoms, such as tremors, hunchbacks, and paralysis of hind limbs, consistent with central nervous system injury (Fu et al., 2019). Mice in this group started to die at 6 dpi and, with 8 days, displayed the shortest median survival time (MST) among the three strains of mice (Fig. 2C, *P*<0.001). In addition, assessment of the behavior score of all groups found that the earliest and most severe neurological symptoms occurred in NCG-hSTAT2^+/+^ mice (Fig. 2D). Clinical sign scoring on 9 dpi was significantly different for NCG-hSTAT2^+/+^ mice compared with C57BL/6J and NCG mice (Fig. 2D, *P*<0.0001).

### Highest viral replication in NCG-hSTAT2^+/+^ mice

**Fig. 3.**
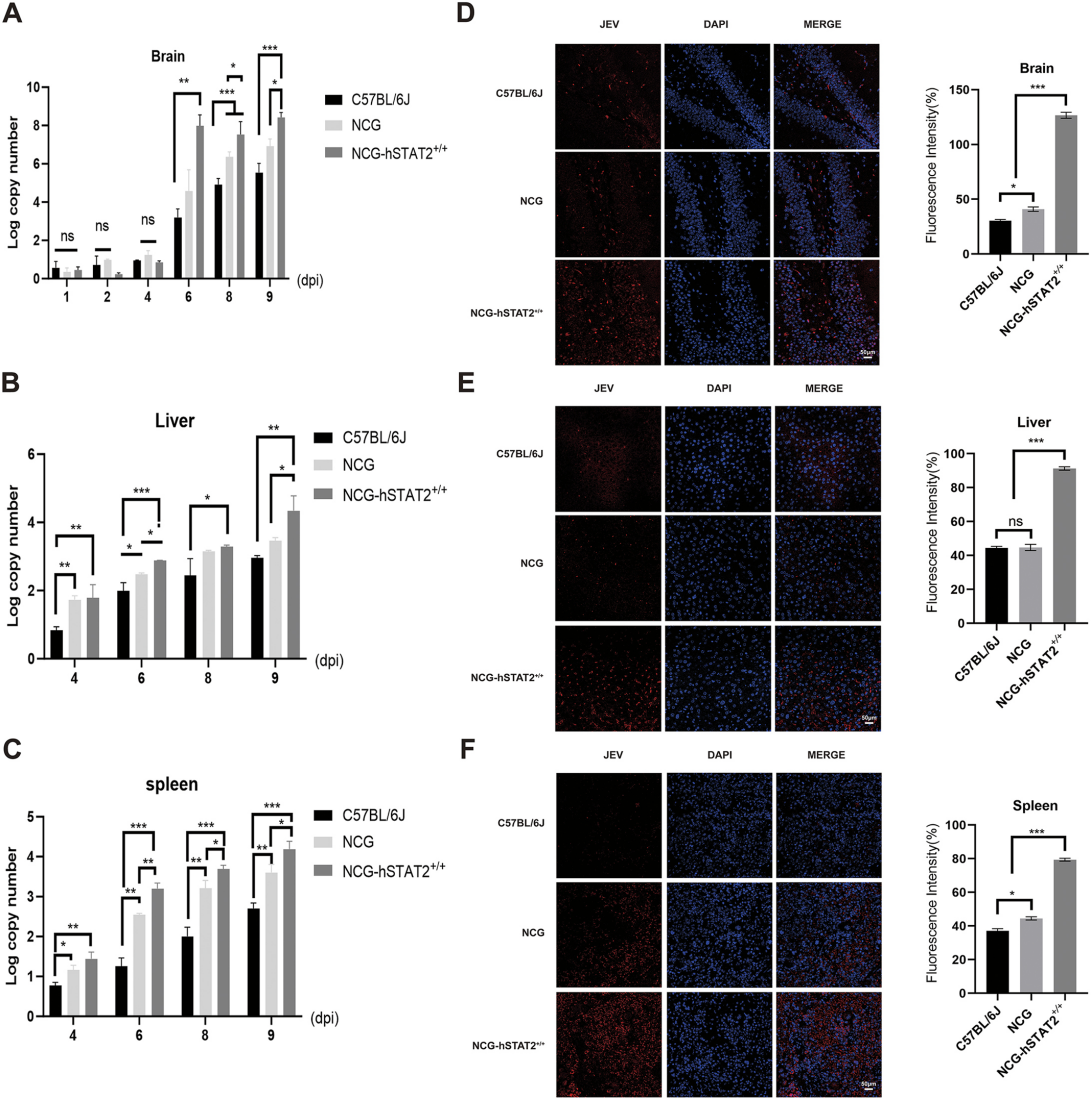
**Brain, liver and spleen JEV load in infected NCG-hSTAT2^+/+^ mice is higher than that of C57BL/6J and NCG mice.** (A-C) Brain, liver and spleen tissue from C57BL/6J, NCG and NCG-hSTAT2^+/+^ mice was collected at different time points after JEV-p3 infection. Viral copy numbers were measured using qRT-PCR. Results indicate that the JEV viral load in all three groups of mice increased over time, with NCG-hSTAT2^+/+^ mice exhibiting a more pronounced increase in JEV viral load. Primers targeting the JEV NS3 gene were designed as detailed in Table S1. Non-infected C57BLL6J mice served as controls, with β-actin as the housekeeping gene. (D-F) Left: Immunofluorescence images of brain (D), liver (E) and spleen (F) tissues from mice as indicated stained for JEV (red; left panels) at day 9 after JEV infection. Nuclei were stained with DAPI (blue) (middle panels); merged images are shown on the right. Right: Bar graphs showing corresponding quantification results. Scale bars: 50 µm. *n*=3. Error bars indicate the + or ±standard error. Statistical significance was assessed by multiple comparisons followed by one-way ANOVA, with *P<0.05, **P<0.01, ***P<0.001. ns, not significant.

To understand the replication of JEV in the central nervous system and peripheral tissues of mice after infection, JEV copy numbers in brain, liver and spleen of the mice were measured. There was no significant difference in the brains in the early stages of viral infection (i.e. 1, 2 and 4 dpi) (Fig. 3). However, at 6, 8 and 9 dpi, viral copy numbers in the brains of NCG-hSTAT2^+/+^ mice were higher than those in NCG and C57BL/6J mice (*P*<0.01) (Fig. 3A). Viral copy numbers were lower in peripheral tissue of liver and spleen at early compared with late stage of infection. From 4 dpi onwards, viral copy numbers in liver and spleen were higher in NCG-hSTAT2^+/+^ mice than in C57BL/6J and NCG mice. Viral loads grew continuously until day 9, and were highest in NCG-hSTAT2^+/+^ mice (*P*<0.001) (Fig. 3B,C). These observations were consistent with the symptoms observed in NCG-hSTAT2^+/+^ mice from 6 dpi onwards. Until death on 9 dpi, NCG-hSTAT2^+/+^ mice showed more severe neurological symptoms compared with mice of the other two groups (Fig. 2D). We, therefore, hypothesized that 9 dpi was the most significant time point regarding differences between NCG-hSTAT2^+/+^ mice and the other two groups of mice and analyzed brain, liver and spleen of all three groups on that day by staining for JEV (Fig. 3D-F). JEV fluorescence intensity was highest in NCG-hSTAT2^+/+^ mice across all organs (*P*<0.001) (Fig. 3D-F, left panels), and quantitative analysis confirmed significant differences between NCG-hSTAT2^+/+^ mice and both C57BL/6J and NCG mice in these tissues (Fig. 3D-F, right panels).

### Expression of IFN signaling molecules and inflammatory cytokines in NCG-hSTAT2^+/+^ mice

**Fig. 4.**
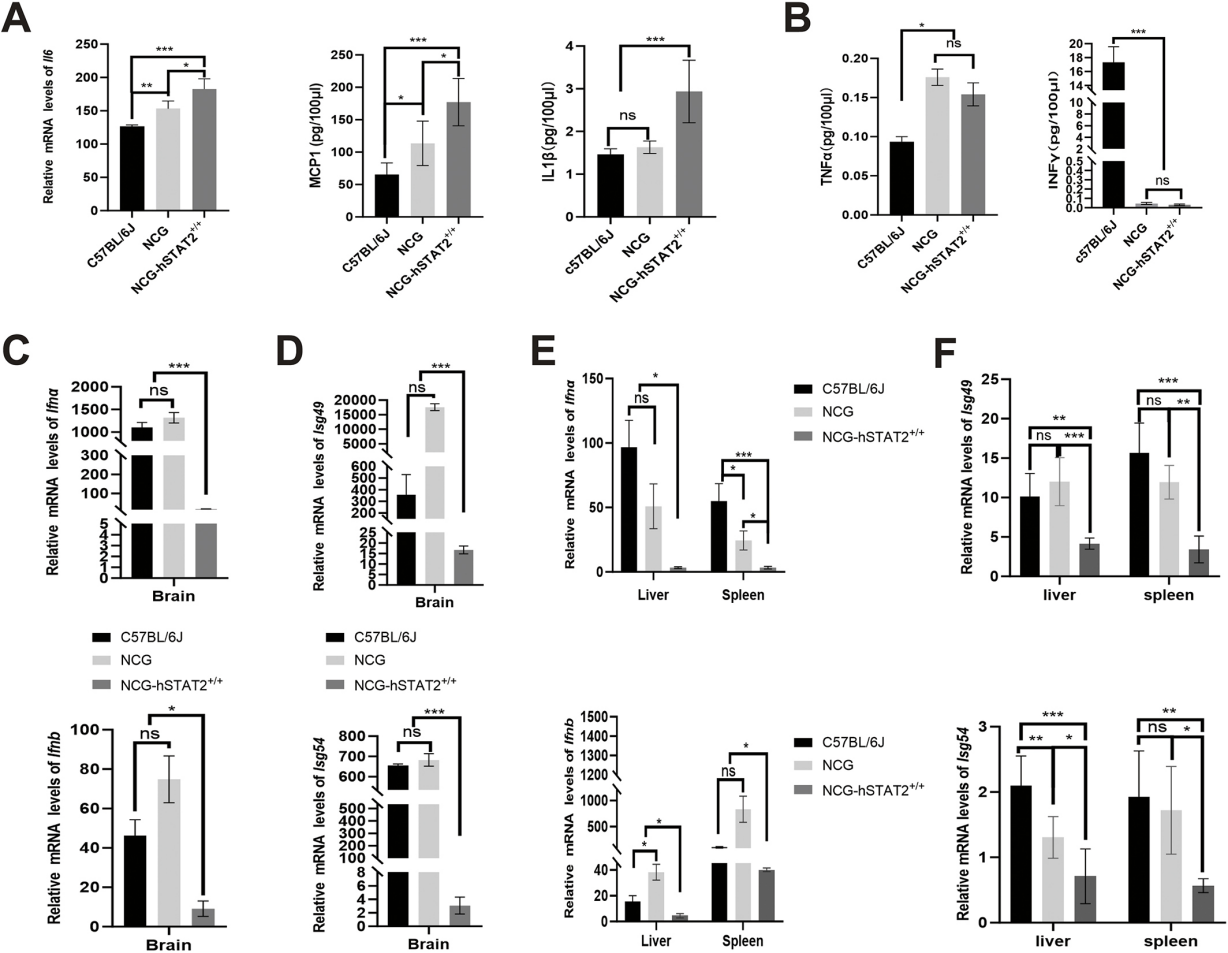
**Brain and peripheral organs of JEV-infected NCG-hSTAT2^+/+^ mice show higher levels of cytokines and lower levels of interferon-related molecules compared with C57BL/6J and NCG mice.** (A) Brain, liver and spleen tissue from C57BL/6J, NCG and NCG-hSTAT2^+/+^ mice was collected at different timepoints (A) Analysis of brain tissue obtained from C57BL/6J, NCG and NCG-hSTAT2^+/+^ mice. Left: Quantification of *Il6* mRNA levels as the fold change relative to the mRNA expression of β-actin. Middle: Quantification of MCP1 protein levels. Right: Quantification of IL1β protein levels. (B) Expression of TNFα and IFNγ protein content in brain tissue of three groups of mice. Protein concentrations of MCP1, IL1β, TNFα, and IFNγ were measured by Luminex multiplex assay. (C,D) Quantification of *Infa*, *Ifnb*, *Isg49* and *Isg54* mRNA expression in brain of three groups of mice by qRT-PCR as the fold change relative to the mRNA expression of β-actin. (E,F) Quantification of *Infa*, *Ifnb*, *Isg49* and *Isg54* mRNA expression in liver and spleen tissues of three groups of mice by qRT-PCR as the fold change relative to the mRNA expression of β-actin. Uninfected C57BL/6J mice served as controls, with β-actin as the housekeeping gene. *n*=4. Error bars indicate the ±standard error. Statistical significance was calculated using ANOVA, with *P<0.05, **P<0.01, ***P<0.001. ns, not significant.

Neuroinflammation is a hallmark of JE, and JEV infection induces the expression of inflammatory factors in the central nervous system (Winter et al., 2004; Tripathi et al., 2021a). To determine whether the expression of inflammatory cytokines in the brain extracts might cause brain injury, we used C57BL/6J, NCG and NCG-hSTAT2^+/+^ mice that had been infected with JEV to examine several inflammatory factors in their brain tissue of on 9 dpi. We found that expression of proinflammatory cytokine IL6 in the brain of NCG-hSTAT2^+/+^ mice was significantly higher than in brains of C57BL/6J and NCG mice, with its levels in NCG mice being significantly higher than in C57BL/6J mice (*P*<0.001) (Fig. 4A). We further detected the protein levels of MCP1, IL1β, TNFα and IFNγ. Levels of MCP1 in the brain of NCG-hSTAT2^+/+^ mice were higher than in C57BL/6J and NCG mice (*P*<0.001) (Fig. 4A). Although total levels of IL1β in all mice groups were lower than total levels of MCP1, brains of NCG-hSTAT2^+/+^ mice also showed the highest expression of IL1β between all groups (Fig. 4A). TNFα levels in brains of NCG-hSTAT2^+/+^ and NCG mice were higher than those of C57BL/6J mice (Fig. 4B). In comparison, NCG-hSTAT2^+/+^ mice showed higher expression of proinflammatory cytokines in the brain, indicating that NCG-hSTAT2^+/+^ mice would exhibit more-severe encephalitis. However, levels of IFNγ in brains of NCG-hSTAT2^+/+^ and NCG mice were lower compared to IFNγ levels in C57BL/6J mice (*P*<0.001) (Fig. 4B).

IFNγ can inhibit the growth of JEV in the central nervous system (Larena et al., 2013), which might be related to the absence of encephalitis-like symptoms in C56BL/6J on 9 dpi. In addition, expression level of IFNα/IFNβ was also closely related to the degree of JEV replication. IFNα/IFNβ bind to their receptor and trigger the JAK-STAT signal pathway, eventually leading to antiviral response and induction of ISGs (Trinchieri, 2010). Therefore, we measured the level of IFNα/IFNβ expression and the induction of antiviral ISGs. Not surprisingly, NCG-hSTAT2^+/+^ mice showed significantly lower mRNA levels of IFNα, IFNβ, ISG49 (officially known as IFIT3) and ISG54 (officially known as IFIT2) in the brains than NCG and C57BL/6J mice on 9 dpi (*P*<0.001) (Fig. 4C,D). In addition, compared with NCG and C57BL/6J mice, NCG-hSTAT2^+/+^ mice also displayed lower mRNA levels of IFNα, IFNβ, ISG49 and ISG54 in liver and spleen (*P*<0.001) (Fig. 4E,F). Therefore, we hypothesized that these mice are unable to effectively combat the virus and, ultimately, exhibit severe paralytic symptoms by 9 dpi. This would likely be due to lower relative expression levels of antiviral genes and interferons in NCG-hSTAT2^+/+^ compared to expressions levels in C57BL/6J mice.

### Extensive activation of microglial cells in brain tissue of JEV-infected NCG-hSTAT2^+/+^ mice

**Fig. 5.**
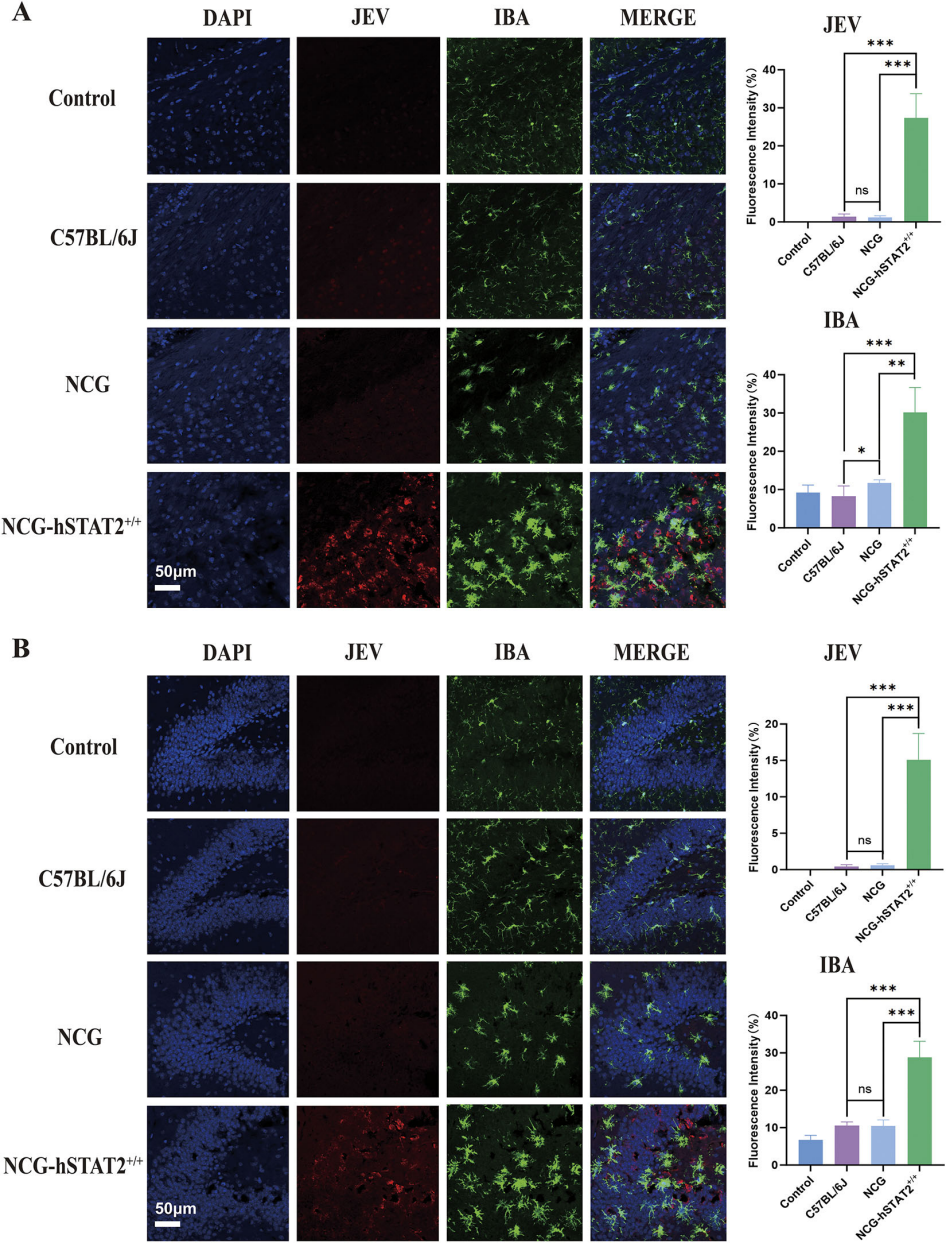
**Infection of most JEV induces the strongest activation of microglia in the brain of NCG-hSTAT2^+/+^ mice.** (A) Left: Immunofluorescence images of active microglia stained for Iba1 (IBA, green) and JEV stained for Iba1 (NS1, red) in cerebral cortex of Control, C57BL/6J, NCG and (NCG-hSTAT2^+/+^ mice. Right: Quantification of fluorescence intensity (in %). (B) Left: Images showing immunofluorescence staining of microglia (IBA) and JEV (NS1) in dentate gyrus of hippocampus of three groups of mice. Right: Quantification of fluorescence intensity (in %). Scale bars: 50 μm. *n*=3. Error bars indicate the +standard error. Statistical significance was calculated using two-tailed unpaired *t*-tests, with *P<0.05, ** *P*<0.01, ***P <0.001; ns, not significant.

To investigate possible encephalitis caused by infection with JEV as well as any differences between groups, we used immunofluorescence staining to observe the characteristics of brain tissues in the three groups of mice on 9 dpi. We found JEV and activated microglial cells within the cortical and hippocampal regions of coronal brain sections (Fig. 5). In cortex tissue of all three groups, a higher degree of JEV infection was accompanied with greater activation of microglial cells (*P*<0.001) (Fig. 5A). In addition, compared with the other two groups, NCG-hSTAT2^+/+^ mice showed the highest JEV load and most-active microglial cells within the hippocampus (*P*<0.001) (Fig. 5B). This indicated that, compared to C57BL/6J and NCG mice, NCG-hSTAT2^+/+^ mice exhibit more-severe JEV infection and inflammation in the brain on 9 dpi.

### Pathological changes in the brain and peripheral tissues of NCG-hSTAT2^+/+^ mice

**Fig. 6.**
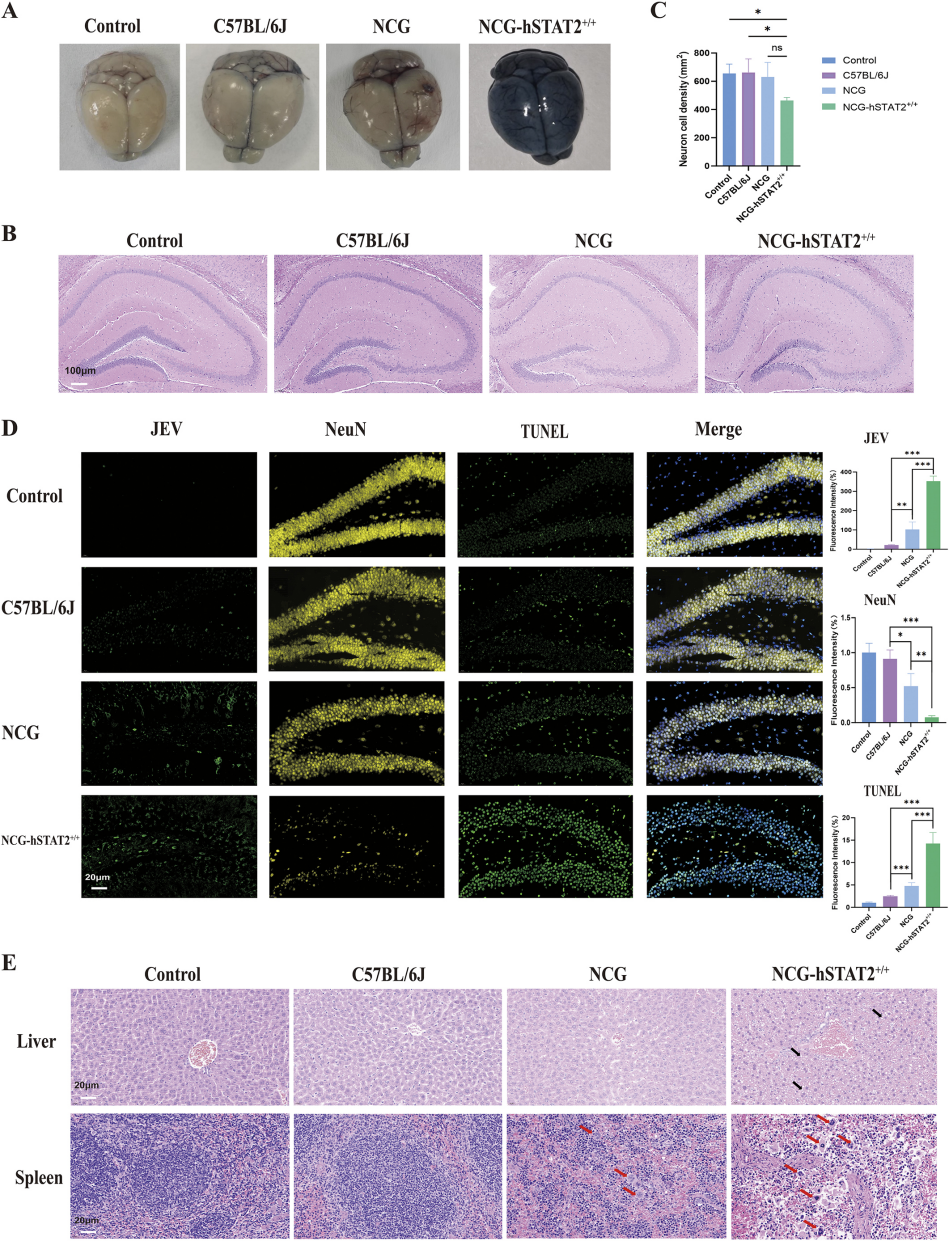
**Pathological changes in the brains and the peripheral organs of the three groups.** (A) Images of the brains obtained from C57BL/6J mice not infected with JEV (Control), C57BL/6J, NCG and NCG-hSTAT2^+/+^ mice showing retention of Evans Blue. (B) Images, showing histopathological changes of the hippocampal area in the brains of mice as described in A. (C) Quantification of neuronal cell density in the hippocampus of the mice as described in B. (D) H&E staining, showing immunofluorescence staining of neuronal cells in the dentate gyrus region. First-left panels: JEV (green). Second-left panels: neuronal cells (yellow). Third-left panels: TUNEL staining (green). Merged images (NeuN, TUNEL and DAPI) are shown on the right. (E) Immunofluorescence images of liver (top) and spleen (bottom) tissue in mice as indicated, showing pathological changes in these peripheral organs. Black arrows indicate fat droplets in the liver of NCG-hSTAT2^+/+^ mice. Red arrows indicate syncytia in the spleen of NCG and NCG-hSTAT2^+/+^ mice. Scale bars: 100 μm (B), 20 μm (D), 20 μm (E). *n*=3. Error bars indicate the standard error. Statistical significance was calculated using two-tailed unpaired *t*-tests., with *P<0.05, **P<0.01, ***P<0.001; ns, not significant.

To examine the changes in the permeability of blood–brain barrier (BBB), Evans Blue stain was performed. The BBB damage in NCG-hSTAT2^+/+^ mice was the most severe, with the deepest staining observed on 9 dpi, and the BBB of C57BL/6J and NCG mice were better (Fig. 6A). Histopathological observation of the brains of each experimental group showed that the density of neurons in the hippocampus of NCG-hSTAT2^+/+^ mice was significantly reduced (*P*<0.05) (Fig. 6B,C). In particular, the neuronal apoptosis in the dentate gyrus region was the most serious. The fluorescence intensity of TUNEL was the strongest in NCG-hSTAT2^+/+^ group, and the fluorescence intensity of JEV was also the strongest (*P*<0.001) (Fig. 6D). It was suggested that most neurons in the brains of NCG-hSTAT2^+/+^ mice were severely infected with JEV, causing severe neurological symptoms. Histopathological results of liver and spleen in peripheral tissues showed that NCG-hSTAT2^+/+^ group had the greatest changes on 9 dpi. Diffuse bleeding and infiltration of fat droplets were observed in the livers of NCG-hSTAT2^+/+^ mice. The spleens of NCG-hSTAT2^+/+^ mice with severe JEV infection showed more giant cells than C57BL/6J and NCG mice (Fig. 6E).

## DISCUSSION

Currently, IFNAR1-KO and AG129 (deficient in both interferon α/β and γ receptors) mice represent well-established flavivirus infection models. However, their applicability for investigating type I interferon-mediated antiviral immunity remains constrained due to the complete ablation of IFN signaling pathways. Wild-type C57BL/6 mice have inherent limitations for JEV interferon escape studies due to robust T-cell responses. Therefore, we established a JEV infection model using STAT2-humanized NCG mice (lymphocyte-deficient background) to investigate JEV pathogenesis and develop therapeutic strategies. The most widely used JEV infection model currently relies on IFNAR1-deficient (IFNAR1-KO) mice (Liu et al., 2022). However, this model primarily manifests lethal peripheral inflammatory responses and systemic pathogenesis without developing neurological symptoms, as JEV fails to establish brain infection in these animals. Conversely, NCG-hSTAT2^+/+^ mice not only showed the pathological changes in peripheral organs (liver and spleen, Fig. 6E), but also revealed higher viral load in brain after infection and exhibited typical neurological symptoms, such as vertical hair, paralysis, hunching, convulsions, stiffness and tremor at an earlier stage (Fig. 2D), similar to the behavioral problems in patients with JEV (Wang et al., 2022; Kalita and Misra., 2000). Brain histopathology revealed microglial activation, reduced neuronal density, and widespread apoptosis due to extensive JEV infection. This is consistent with the histological features of fatal cases of JE in humans (Turtle and Solomon, 2018), which indicates that NCG-hSTAT2^+/+^ mice closely recapitulate the characteristic histopathological features of human JEV infection.

The other most used JEV infection model-AG129 mice, a study using AG129 mice demonstrated peripheral characteristics after intraperitoneally infection with JEV vaccine, SA14-14-2, at a low dose (10 PFU/0.1 ml) (Calvert et al., 2014). This model exhibited solely peripheral manifestations without developing neurological symptoms characteristic of encephalitis. Furthermore, it demonstrated 100% mortality within 5 days post-infection, indicating rapid disease progression and an abbreviated clinical course. Given its acute onset and short disease duration, this model might present limitations for evaluating vaccines and antiviral therapeutics against JEV due to the constrained experimental window. In contrast, NCG-hSTAT2^+/+^ mice inoculated with a low viral dose (10³ PFU) of JEV-p3 exhibited hallmark neurological symptoms with delayed mortality onset (7–11 days post-infection). The protracted disease course in this model provides an extended therapeutic window, enabling evaluation of antiviral strategies and pathogenesis studies. In addition, several studies have established JEV-induced encephalitis murine models utilizing clinical isolates with elevated viral inoculation. For instance, intracerebral and intraperitoneal inoculation of AG129 mice with 0.5×10^5^ PFU of the Indian clinical JEV strain P20778 successfully recapitulated the hallmark features of neurotropic encephalitis (Siddqui et al., 2022). However, this AG129 mouse model with the absence of interferon immunoregulatory pathway, required a higher viral inoculum than the NCG-hSTAT2^+/+^ mice (10³ PFU). The mice showed severe clinical pathological symptoms and 100% mortality (within 4-8 dpi) in intraperitoneal (IP) and intra dermal (ID) routes of JEV infection. This rapid disease progression significantly constrains the experimental window for therapeutic intervention. In our study, the NCG-hSTAT2^+/+^ mice were inoculated via footpad injection with 10³ PFU of JEV to simulate natural mosquito-borne transmission, establishing 100% mortality occurring between 7-11 days post-infection to guarantee sufficient experimental period. Meanwhile, key inflammatory mediators (MCP1, IL1β, IL6, TNFα) were significantly elevated in NCG-hSTAT2^+/+^ mice during viral neuroinvasion, likely driving encephalitis pathogenesis. Reduced interferon levels correlated with high viral loads and severe symptoms. Thus, NCG-hSTAT2^+/+^ mice with complete interferon immunoregulatory pathway model effectively recapitulates human JEV infection progression. At a markedly lower infectious dose (10^3^ PFU), the adult NCG-hSTAT2^+/+^ model recapitulated the key pathological features observed in 3–4-week-old C57BL/6 mice infected with the mouse-adapted JEV-S3 strain (10^6^ PFU), including robust microglial activation, neuronal apoptosis, and significant induction of inflammatory cytokines (Tripathi et al., 2021b).

Two other studies found that infection of hSTAT2-KI mice with ZIKV did not only recapitulate the extreme vulnerability and lethality of mice lacking the interferon receptor (IFNAR1^−/−^; Lazear et al., 2016) or STAT2 (STAT2^−/−^ mice; Tripathi et al., 2017), but also reflect the cell-extrinsic antiviral effects of IFN signaling (Yang et al., 2024). These hSTAT2-KI mice, which have human STAT2 replacing mouse STAT2, developed an intact IFN immunocompetent model of ZIKV infection and pathogenesis after peripheral inoculation. This model more closely recapitulates the features of human infection (Gorman et al., 2018). Our NCG-hSTAT2^+/+^ mouse model uniquely preserves IFN pathway integrity due to expression of hSTAT2, thereby maintaining this signaling cascade (Fig. 1G). Compared to knockout models, this genetically humanized mouse model recapitulates human immune responses and clinical manifestations, making it superior for viral pathogenesis studies. Our findings demonstrate that JEV-infected NCG-hSTAT2^+/+^ mice model encephalitis far better compared to existing models (Fig. 4, Figs S1 and S2). These data collectively delineate the dynamic interplay between JEV and the IFN system. These mice lack adaptive immunity (T/B/NK cells) while retaining intact humanized IFN signaling, making them ideal for studying virus-induced IFN responses. Notably, STAT2-humanized mice developed more severe encephalitis than C57BL/6J or IFN receptor-deficient mice, suggesting JEV employs a STAT2 degradation mechanism similar to that of DENV and ZIKV (Ashour et al., 2009, 2010; Grant et al., 2016). In Fig. S2A, western blotting revealed that JEV infection induces degradation of hSTAT2 protein in NCG-hSTAT2^+/+^ mice. Further validation of these findings is warranted.

While our study has provided valuable insights, it still has some limitations. This is due to the genetic, anatomical, physiological, and immunological differences between mice and humans. For example, due to the lack of a complete immune system NCG mice might not be able to present certain immune responses. NCG-hSTAT2^+/+^ mice retain an intact type I interferon axis yet are congenitally deficient in T, B, and NK lymphocytes. Therefore, the model is restricted to interrogating JEV–IFN interactions and does not recapitulate the complete adaptive immune response observed in immunocompetent hosts. However, this model with a humanized immune system has emerged as a powerful tool for studying infectious diseases (Coronel-Ruiz et al., 2020; Zompi and Harris, 2012; Mota and Rico-Hese, 2011). Therefore, further humanization of the immune system of NCG-hSTAT2^+/+^ mice, such as a gene-immune system double-humanized mouse model, may better reproduce the key biological processes involved in the development and progression of JEV-induced diseases, thus providing a better basis for clinical research. For example, by transplanting human hematopoietic stem cells into NCG-hSTAT2^+/+^ mice, we can develop a dual – genetic and immune – humanized model that is well suited for investigating viral infections.

## CONCLUSIONS

**Fig. 7.**
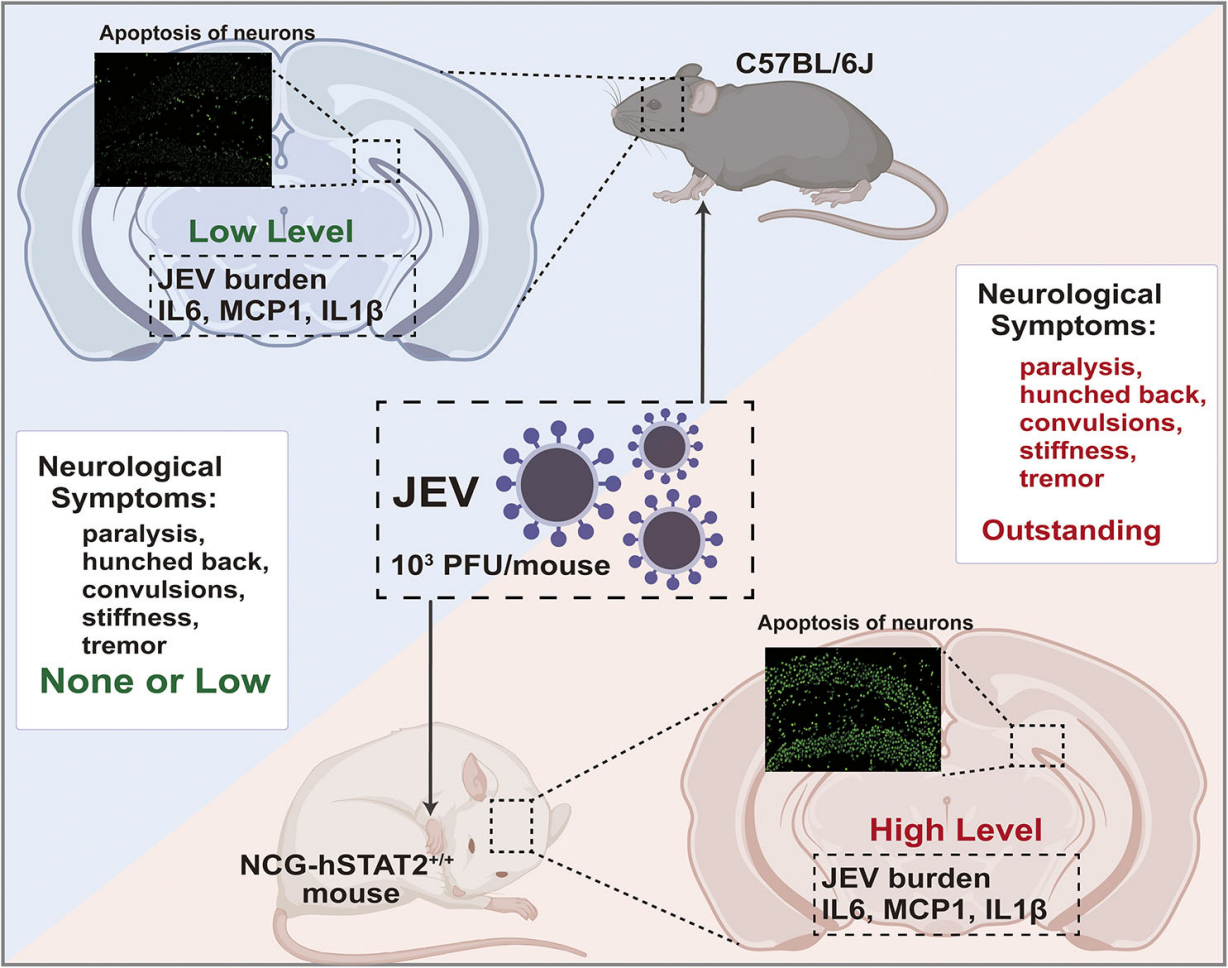
**NCG-hSTAT2**^**+/+**^ **mice represent a novel model of JEV infection.** NCG-hSTAT2^+/+^ mice challenged with a low dose of JEV develop severe neurological signs – paralysis, hunched back, convulsions and tremor – mirroring the human clinical phenotype. Their brains display robust microglial activation, widespread neuronal apoptosis, and high levels of JEV and inflammatory cytokines (IL6, MCP1, IL1β), validating the model for studying JEV neuropathogenesis.

Mice expressing the human STAT2 gene, a key molecule in the IFN pathway, have been created to allow this pathway to remain intact and facilitate JEV infection. When infected with a low dose of JEV, this humanized mouse model displays a higher viral burden, produces higher levels of proinflammatory cytokines, and shows typical microglia activation and neuronal apoptosis in the brain. Especially, typical neurological symptoms, such as piloerection, paralysis, hunchback and tremor at an early stage can be observed. This suggests that NCG-hSTAT2^+/+^ mice exhibit higher susceptibility to JEV and more severe neurological symptoms, which is consistent with clinical manifestations in human patients (Fig. 7). Thus, NCG-hSTAT2^+/+^ mice can serve as an animal model for studying JEV infection and disease pathogenesis, and may be used as a preclinical model to evaluate the safety and efficacy of therapeutic strategies.

## MATERIALS AND METHODS

### Animals and ethics approval

NCG-hSTAT2^+/+^ mice are Stat2-humanized mice constructed on the background of the severely immunodeficient NCG strain, and were created by GemPharmatech (Nanjing, China) using CRISPR/Cas9 technology (Fig. 1A).

40 NCG mice (Strain NO. T001475) were purchased from GemPharmatech (SCXK(Su)2023-0009, Nanjing, China). 70 C57BL/6J and 40 NCG-hSTAT2^+/+^ mice were provided from the Laboratory Animal Center of the Air Force Medical University [licence no.: SCXK(Shan)2019-001, Shaanxi, China]. At age 6–8 weeks, mice were equally divided by sex, distributed across three groups and maintained in a barrier environment at the Laboratory Animal Center of the Air Force Medical University [licence no.: SYXK(Shan)2019-001]. The ambient temperature was 23–25°C, the relative humidity was 40–60%, and a 12/12-h light–dark cycle was used. Cages, bedding material, food and drinking water of all mice were subjected to high-temperature treatment and autoclaved to ensure sterility. Animals were provided *ad libitum* access to food and water. All animal experiments complied with the ARRIVE guidelines (https://arriveguidelines.org/) and were carried out in accordance with the UK Animals (Scientific Procedures) Act, 1986, and associated guidelines, EU Directive 2010/63/EU for animal experiments. All animal experimental protocols were approved by the Institutional Animal Care and Use Committee of the Air Force Medical University (No. 20200602).

### Virus

The JEV-p3 strain was obtained from the Pathogenic Microbiology Laboratory of the Air Force Medical University and propagated in BHK-21 cells that were cultured and maintained in Dulbecco's Modified Eagle Medium (DMEM) supplemented with 2% fetal bovine serum, penicillin (100 U/ml), and streptomycin (100 U/ml). Virus titers were determined by a cytopathic assay using BHK21 cells, and aliquots of virus stocks were stored at −80°C.

### Infection

To determine the survival rate, C57BL/6J mice (*n*=30) were inoculated through their hindlimb foot pads with JEV-p3 at 10^1^–10^6^ plaque-forming units (PFU)/mouse. Based on these results, 10^3^ PFU was selected as an appropriate dose to inoculate three groups of mice, i.e. NCG, C57BL/6J and NCG-hSTAT2^+/+^ mice (*n*=30 mice/group). The initial body weights of the mice were measured to record symptoms and body weight changes each day following infection. Meanwhile, tissues were collected at 1, 2, 4, 6, 8 and 9 days post infection (dpi) to detect the viral burden using quantitative real-time PCR (qRT-PCR). For enhanced safety, all mouse infection experiments with JEV were performed according to Animal Biosafety Level 2 (A-BSL2).

### Clinical sign scoring of Japanese encephalitis

The system used to score signs of Japanese encephalitis (JE) in mice was as follows. Score 0: no restriction in movement, no piloerection (i.e. temporarily raised hair on the skin surface), no body stiffening, no hindlimb paralysis. Score 1: no restriction in movement, no body stiffening, no hindlimb paralysis but piloerection. Score 2: no movement restrictions, slow movement, arching back. Score 3: limited movement, piloerection, mild muscle stiffness, mild convulsions, slight hindlimb extension but no hindlimb paralysis. Score 4: limited movement, piloerection, stiff body, hindlimb paralysis. Score 5: limited movement, hair erection, body stiffness, hindlimb paralysis, tremor, death.

### RNA extraction and qRT-PCR

Approximately 100 mg of brain, liver or spleen tissue samples collected from mice at different time points were suspended in 1 ml ice-cold saline solution and homogenized separately. Thereafter, aliquots of 300 µl were centrifuged (5000 rpm, 5 min using a 5810R Eppendorf centrifuge) and pellets lysed in 900 µl Trizol for RNA extraction. Total RNA was extracted using a commercial RNA extraction kit (cat. no. R6834-02; Omega Bio-tek, Norcross, GA, USA). RNA concentration was measured with a microplate reader and adjusted to 500 ng/µl. RNA was reverse-transcribed into cDNA according to the manufacturer's instructions (Prime Script RT Master Mix, cat. no. RR036A; Takara Dalian, China), and reverse transcription conditions were set at 37°C for 15 min and 85°C for 15 s. qRT-PCR was performed using a TaKaRa kit (SYBR Premix Ex Taq II, cat. no. RR820A; Takara). The qRT-PCR reaction system (20 µl) comprised 10 µl SYBR Premix Ex Taq II, 1 µl each of upstream and downstream primers, 0.4 µl of ROX reference dye (50×), 1 µl of cDNA template, and 6.6 µl of double-distilled H_2_O. qRT-PCR reaction conditions were: pre-denaturation at 95°C for 30 s, followed by 40 cycles at 95°C/15 s and 40 cycles at 60°C/30 s (fluorescence measurement). Cycle threshold (Ct) values for each sample were measured; each group of sample was tested three times, and each test was performed three times. Final Ct values are expressed as the average value. Then, the 2−ΔΔCt method was applied to quantify relative mRNA expression levels. Expression of JEV NS3, IL6, IFNα, IFNβ, ISG49, ISG45 and ISG56 are presented as the relative fold expression after normalization to the housekeeping gene β-actin. Primer sequences are listed in Table S1 (Patil et al., 2021).

NCG and NCG-hSTAT2^+/+^ mice were administered 1 mg/ml of Poly(I:C) (HMW) (Invivogen, cat. no.: tlrl-pic) intraperitoneally, and spleens were harvested 6 h later. Total RNA was extracted from spleen tissue homogenates. qRT-PCR was performed as described above. ISGs were detected by using primers targeting mouse genes *Irf7*, *Oas1* or *Ifnb1* (see Table S1) (Gorman et al., 2018).

### Blood–brain barrier permeability assay

At 9 dpi three mice from each of the three groups were administrated 0.2 ml of 1% Evans Blue dye through the tail vein. After 2 h of incubation, mice were euthanized and perfused with PBS. Brains were then collected for observation.

### Hematoxylin and eosin (H&E) staining and immunofluorescence analysis

Brain, liver and spleen tissues from mice at 9 dpi were used for histopathological examination. Tissues were washed with pre-cooled PBS and fixed in 4% paraformaldehyde. After 48 h, tissues were dehydrated, embedded in paraffin and cut into sections of 4 μm. Tissues were H&E stained and any pathological damage was investigated under a light microscope. After anesthesia, mouse brain tissue was first perfused with saline solution through the heart, followed by perfusion with 4% paraformaldehyde (Servicebio, Wuhan, China). Brain tissue was then extracted and stored in 4% paraformaldehyde for 24 h for fixation, followed by dehydration though a 25% and 30% sucrose gradient. The brain tissues were cut at −20°C into coronal sections using a microtome (CM1950 S, Leica, Shanghai, China) at a thickness of 20 μm, naturally dried and fixed, and frozen at −20°C. Brain slices were deparaffinized and washed together with frozen sections in PBS (pH 7.4) for 5 min and then permeabilized with 0.2% Triton 20 for 10 min before processing for immunofluorescence labeling. Sections were incubated in 2% BSA for 1 h, followed by overnight incubation at 4°C with primary antibodies against allograft inflammatory factor 1 (IBA1, officially known as AIF1) (IBA, 1:500, cat. no.: Ab178846, Abcam), neuron-specific nuclear protein (Neun, 1: 500, cat. no.: ABN78, Millipore, Burlington, MA, USA), Japanese encephalitis virus NS1 [(JEV NS1), 1: 500, GT1410, cat. no.: GTX633820, GeneTex, USA). The next day, slices were washed three times in PBS and sections incubated with corresponding secondary antibodies (DyLight 594 and DyLight 488, cat. no.: GTX213110-05 and GTX213110-04, 1:500, Genetex, Irvine, CA, USA) in the dark at 20–25°C for 2 h. Sections were then washed thrice in PBS for 5 min each and protected from light. Nuclei were stained with DAPI (10 μg/ml, Leagene, Beijing, China) for 10 min, and mounted using glycerin gelatin. Imaging was then performed using a confocal laser scanning microscope (LSM900, Zeiss, Germany). Brains were strained using the Fluorescein (FITC) TUNEL Cell Apoptosis Detection Kit (cat. no.: G1501-50T, Servicebio, China).

### Detection of cytokines in brain homogenate by using the Luminex multiplex assay

JEV-infected mice (*n*=3-4/group) were euthanized on days 3, 5, 7 and 9 dpi. Half of their brains were homogenized in 800 µl radioimmunoprecipitation assay (RIPA) buffer (cat. no.: R0010, Solarbio, Beijing, China) on ice, lysed at 4°C for 1 h and centrifuged at 12,000 rpm for 10 min. Supernatant protein was isolated and quantified by bicinchoninic acid (BCA) assay. Luminex assays were performed according to the manufacturer's protocol using a Mouse Premixed Multi-Analyte Kit (R&D Systems, Minneapolis, MN, USA, cat. no.: LXSAMSM-05, Lot: L149522) to quantify cytokine levels of MCP1, IL1β, TNFα, IFNγ and IL6 in brain lysates. Cytokines levels detected in lysates were analyzed by using standard curves obtained from determined protein concentrations.

### Western blotting

Expression of the human *STAT2* gene in heart, liver, spleen, lung, kidneys and brain of NCG-hSTAT2^+/+^ and NCG mice was analyzed using western blotting. In brief, total proteins were separated using RIPA lysis buffer (Beyotime, Shanghai, China) and subjected to 10% sodium dodecyl sulfate−polyacrylamide gel electrophoresis (SDS−PAGE). Next, proteins were transferred to PVDF membranes (Millipore, Merck, Darmstadt, Germany) in skimmed milk, followed by incubation with anti-STAT2 (cat. no.:51075-2-AP, Proteintech; 1:500), β-actin (cat. no.: BS00244-T, Bioworld; 1:1000) and secondary antibodies (ab205718, ab205719; Abcam) were used. Western blotting was performed using the enhanced chemiluminescence kit (cat. no.: WBKLS0100, Millipore, Beyotime).

### Statistical analysis

GraphPad Prism 9 was used for statistical analysis. The data were analyzed using two-tailed unpaired *t*-tests and one-way ANOVA. Differences between three or more conditions with one independent variable were analyzed by one-way analysis of variance. Significance: *P<0.05, **P<0.01 and ***P<0.001. Image J software was used to analyze immunofluorescence results and determine the mean gray value.

## Supplementary Material

10.1242/dmm.052431_sup1 Supplementary information
