# Supplementary material for: Characteristics of Japanese encephalitis virus infection in NCG-hSTAT2+/+ mice: a novel model for studying neurological symptoms and immune response
Source: Dis Model Mech. 2025 Nov 18;18(11):dmm052431. doi: 10.1242/dmm.052431 (PMC12661643; doi:10.1242/dmm.052431)
Supplement: Supplementary information [file dmm-18-052431-s1.pdf]

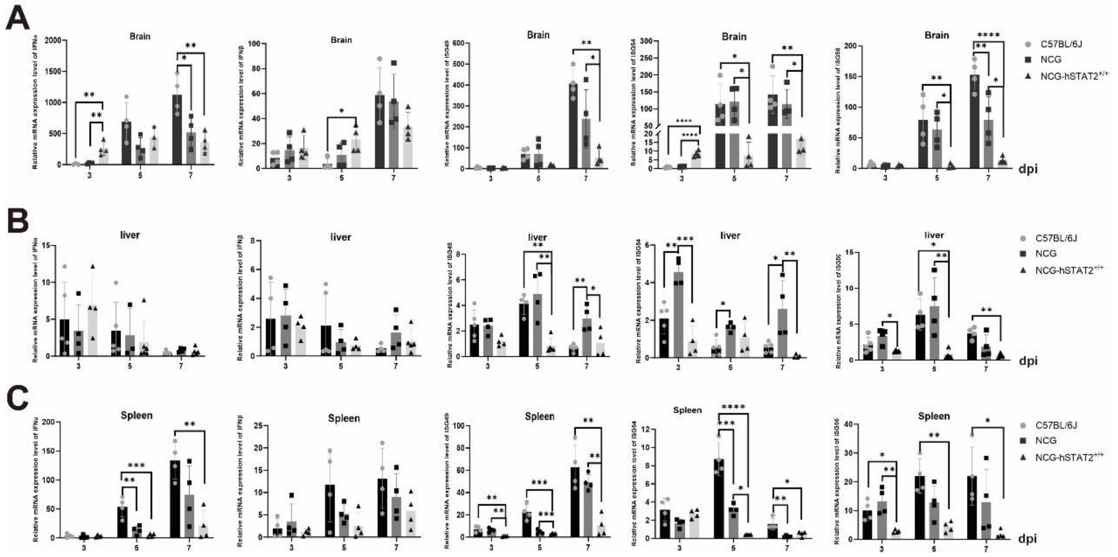

Fig. S1. Expression of interferon and interferon-stimulated genes in the JEV-infected mouse brain and peripheral organs.

(A) C57BL/6J, NCG and NCG-hSTAT2$^{+/+}$ mice (6-8 weeks old, n=4) had inoculated hindlimb foot pads with 20 µl JEV-p3 (5×10^{4} PFU), and brain tissues were harvested from mice at 3, 5, 7 dpi. RNA was extracted from the tissue, and qRT-PCR was performed to study gene (IFNα, IFNβ, ISG49, ISG54 and ISG56) expression. β-action mRNA level was used for normalization. (B-C) They shows mRNA expression in liver and spleen tissues of three groups of mice by qRT-PCR. Uninfected C57BL/6J mice served as controls. Statistical significance *$p < 0.05$; **$p < 0.01$; ***$p < 0.001$ was assessed by multiple comparisons following one-way ANOVA, n=4.

Disease Models & Mechanisms • Supplementary information

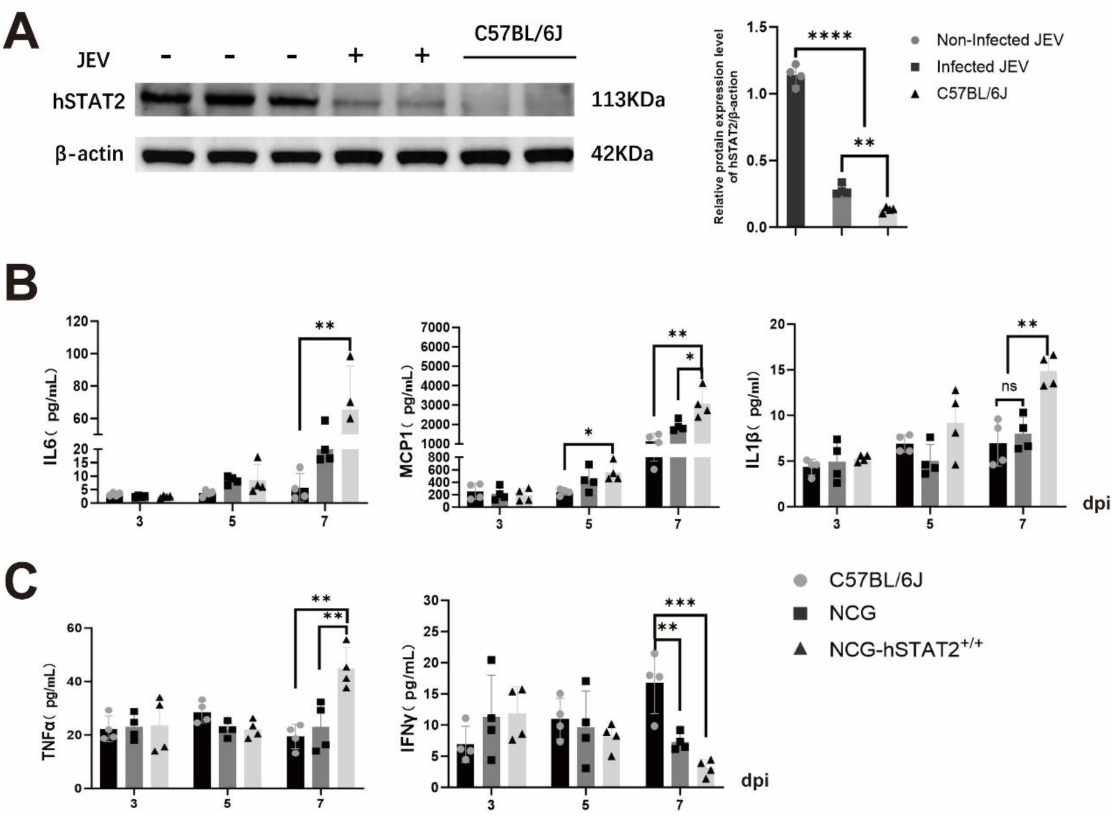

Fig. S2. Expression of human STAT2 and inflammatory cytokine levels in the JEV-infected mouse brain.

(A) NCG-hSTAT2$^{+/+}$ mice (6-8 weeks old, n=4) had inoculated hindlimb foot pads with 20 μl JEV-p3 (5×10^4 PFU), and brain tissues were harvested from mice at 8 dpi. Uninfected NCG-hSTAT2$^{+/+}$ and C57BL/6J mice served as controls. Human STAT2 expression levels were analyzed by Western blotting. The histogram on the right displayed the quantified data. (B-C) C57BL/6J, NCG and NCG-hSTAT2$^{+/+}$ mice (6-8 weeks old, n=4) had inoculated hindlimb foot pads with 20 μl JEV-p3 (5×10^4 PFU), and brain tissues were harvested from mice at 3, 5, 7 dpi. Protein lysates were prepared from the tissue, concentration levels of IL6, MCP1, IL1β, TNFα, and IFNγ were measured by Luminex assay. Statistical significance *$p < 0.05$; **$p < 0.01$; ***$p < 0.001$ was assessed by multiple comparisons following one-way ANOVA, n=4.

Disease Models & Mechanisms • Supplementary information

Table S1. Primer sequence information.

**Primer name**	**Primer sequence** (5′ to 3′)
hSTAT2 5′	forward: AATGACCAGCAGAGAACGGGT
	reverse: AGAACTGGCTCTCCTTGTTCCAA
hSTAT2 3′	forward: GCATCGCATTGTCTGAGTAGGTG
	reverse: TGTCTCTGAGACCCTGTGTGCTTAG
mSTAT2 (WT)	forward: AATGACCAGCAGAGAACGGGT
	reverse: TGTCTCTGAGACCCTGTGTGCTTAG
JEV（NS3）	forward: AGACAAGCAGATCAACCACCATT
	reverse: CCCTCCAATAGAGCCAAAGTCC
IL6	forward: TGGGAAATCGTGGAAATGAG
	reverse: CTCTGAAGGACTCTGGCTTTG
IFNα	forward: TGTCTGATGCAGCAGGTGG
	reverse: AAGACAGGGCTCTCCAGAC
IFNβ	forward: TCCAAGAAAGGACGAACATTCG
	reverse: TGAGGACATCTCCCACGTCAA
ISG49	forward: GCCGTTACAGGGAAATACTGG
	reverse: CCTCAACATCGGGGCTCT
ISG54	forward: GGGAAAGCAGAGGAAATCAA
	reverse: TGAAAGTTGCCATACAGAAG
ISG56	forward: ATGGAGCACGGACTCAGGA
	reverse: TCACACACGACATTGACGGC
Oas1	forward: CAGCTCCAAGAAAGGACGAAC
	reverse: GGCAGTGTAACTCTTCTGCAT
IFNb1	forward: GAGACTGGCTATTGGGGGAG
	reverse: GACCGAAATGCTTCCAGGG
IRF7	forward: GGCTGTATTCCCCTCCATCG
	reverse: CCAGTTGGTAACAATGCCATGT
β-actin	forward: AATGACCAGCAGAGAACGGGT
	reverse: AGAACTGGCTCTCCTTGTTCCAA

hSTAT2, human STAT2; mSTAT2, mouse STAT2.

Disease Models & Mechanisms • Supplementary information
